# Contrast-induced Nephropathy Following Percutaneous Coronary Intervention at a Tertiary Cardiac Center in Nepal

**DOI:** 10.7759/cureus.3331

**Published:** 2018-09-18

**Authors:** Amrendra Mandal, Mukesh S Paudel, Paritosh Kafle, Mazin Khalid, Bikash Bhattarai, Rajan Kanth, Arun Maskey, Jenny Lamicchane, Neetu M Ray, Dikshya Sharma, Shristy Gautam, Vijay Gayam

**Affiliations:** 1 Internal Medicine, Interfaith Medical Center, Brooklyn, USA; 2 Internal Medicine / Gastroenterology, Lumbini City Hospital, Butwal, NPL; 3 Internal Medicine / Pulmonology, Interfaith Medical Center, New York, USA; 4 Gastroenterology, Carilion Clinic, Roanoke, USA; 5 Cardiology, Sahid Gangalal National Heart Center, Katmandu, NPL; 6 Internal Medicine, St. John Riverside Hospital, Yonkers, USA; 7 Neuropediatrics, Practitioner, Flushing, USA; 8 Internal Medicine, Maimonides Medical Center, Brooklyn, USA; 9 Dentistry, KIST Medical College, Kathmandu, NPL

**Keywords:** percutaneous coronary intervention, coronary artery disease, contrast induced nephropathy, hemodialysis

## Abstract

Background

Contrast-induced nephropathy (CIN) is one of the leading causes of morbidity and mortality including increased financial burden in high risk patients undergoing percutaneous coronary intervention (PCI).

Methods

This is an observational prospective study. We aimed to study the incidence of CIN in Nepalese populations and compare the outcome to international reprinted values with coronary artery disease (CAD) undergoing PCI. All consecutive patients with CAD undergoing PCI between February 2010 and July 2010 were enrolled in the study.

Results

One hundred fifty-two patients were enrolled in the study during six months period. Twenty (13.20%) patients developed CIN following PCI. Out of them 70% were diabetics and 30% were non-diabetics. Mean age of patients was 58.5 ± 23 years; male:female ratio was 2.7:1. Mean contrast volume injected was 160.3  ±  78.3  mL. Diabetic patients 21.8% (14/64) had significant CIN compared to non-diabetic patients 6.8% (6/88) following PCI (<0.01).

Conclusions

CIN is a common complication following PCI especially in diabetics. Despite the use of iodinated material we had similar incidence of CIN comparing the incidence of CIN among various radiocontrast compounds used to visualize vessels. None of the patients received hemodialysis as compared to available studies and there was no observed mortality.

## Introduction

Contrast-induced nephropathy (CIN) refers to the reversible form of acute renal failure that results in secondary to contrast exposure. CIN is a serious complication of percutaneous coronary intervention (PCI), and is associated with considerably increased morbidity, including the need for short-term hemodialysis, extended hospitalization, and permanent impairment of renal function [1]. Most importantly, the development of CIN is independently associated with increased in-hospital and long-term mortality [2]. We aimed to study the incidence of CIN in patients undergoing PCI at a tertiary cardiac center in Nepal.

## Materials and methods

We prospectively enrolled 152 consecutive patients (age ≥ 18 years) with the diagnosis of acute coronary syndrome or the worsening symptoms in patients with known history of coronary artery disease (CAD) who were admitted to undergo PCI, between February 2010 and July 2010 at Sahid Gangalal National Heart Center, Kathmandu, Nepal. Study was approved by the institutional review board (IRB) at National Academy of Medical Sciences (NAMS). Acute coronary syndrome diagnosis was based on standard criteria of chest pain, ischemic changes in EKG and elevated cardiac enzymes. The exclusion criteria included pregnancy, contrast media (CM) allergy, end stage kidney disease and unwillingness to give consent. PCI was performed by interventional cardiologists using a standard technique. All patients undergoing PCI received either Ioversol or Iohexol. The volume of contrast media was calculated based on total volume used for every single procedure. Pre- and post-procedural serum creatinine including baseline labs were obtained before the PCI at 24 hours, 48 hours, and 72 hours after the PCI.

Primary outcome of the study was to assess the incidence of CIN in CAD patients undergoing PCI. CIN was defined as 25% relative increase, or a 0.5 mg/dL (44 µmol/L) absolute increase in serum creatinine (SCr) between 48 and 72 hours of contrast exposure, in the absence of an alternative explanation [3]. Secondary outcome was to assess the risk factors for the development of CIN.

Statistical analysis

The data was entered into Microsoft excel and analyzed using SPSS 17 (IBM Corporation, Armonk, NY). The continuous data was expressed as mean and standard deviation while categorical data was expressed as number (percentage). Statistical tests like chi-square test and t-test were used to assess the difference between parameters. Statistical significance was taken as p < 0.05 level.

## Results

**Figure 1:**
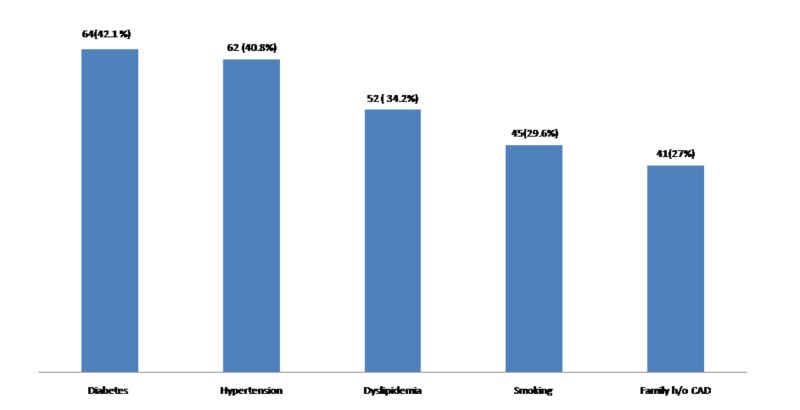
Risk factors and co-morbidities.

A total of 152 patients were eligible for the present study (mean age 58.5 ± 23 years, mean contrast volume 160.3 ±  78.3  mL), and males were predominant group participated in the study with male:female ratio of 2.7:1. Most common co-morbidities for CAD were diabetes and hypertension, as depicted in Figure 1. The baseline characteristics are displayed in Table 1.

**Table 1:** Baseline characteristics of the study populations. ACS: Acute coronary syndrome

Factors	N (%) or mean ± SD
Age (years)	58.5 ± 23
Male	111 (73%)
Systolic BP (mm of Hg)	127 ± 47
Baseline creatinine (mg/dL)	1.06 ± 32
ACS STEMI NSTEMI UNSTABLE ANGINA	76 (37.5%) 27 (17.76%) 27 (17.76%)
Contrast volume (mL)	160.3 ± 78.3

Incidence of CIN

**Figure 2:**
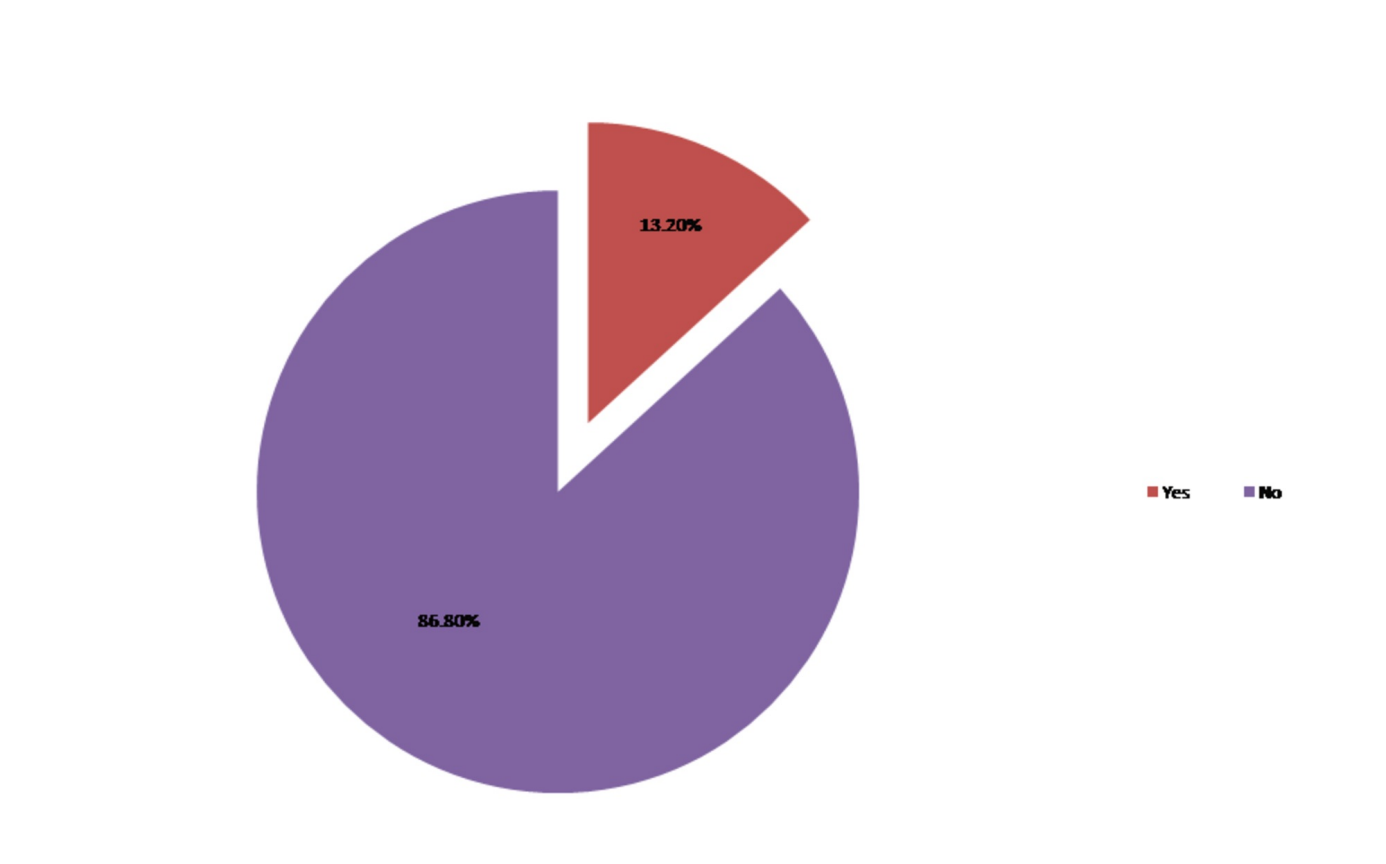
Incidence of contrast-induced nephropathy (CIN) in a study population.

**Figure 3:**
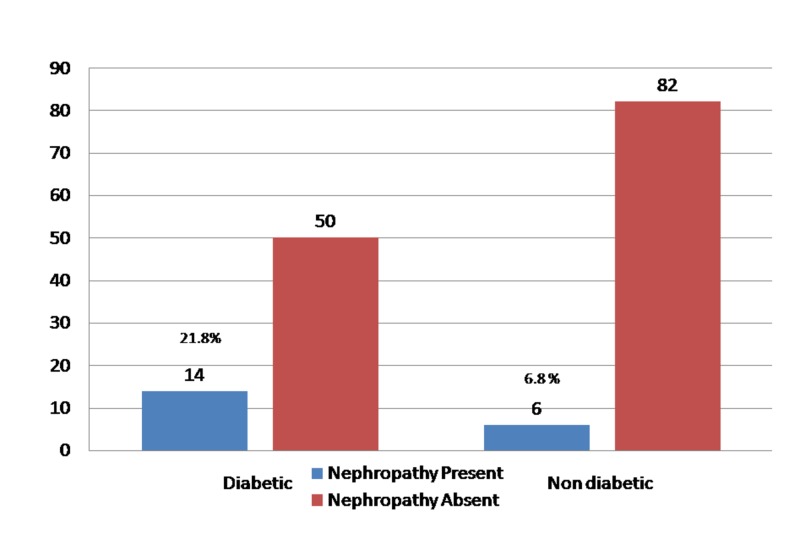
Contrast agent nephropathy among diabetics and non-diabetics.

Twenty (13.20%) patients developed CIN following PCI as depicted in Figure 2 and out of them 70% were diabetics and 30% were non-diabetics. However, when patients were re-stratified based on the presence of diabetes, CIN was significantly elevated in diabetics 21.8% (14/64) compared to non-diabetics 6.8% (6/88) following PCI as depicted in Figure 3.

There was significant rise in post-procedural creatinine between 48 and 72 hours when comparing baseline creatinine between diabetic and non-diabetic patients as depicted in Table 2.

**Table 2:** Contrast-induced nephropathy (CIN) in diabetic and non-diabetic patients. PCI: Percutaneous coronary intervention

Risk factors of CIN (N = 152)	Non-diabetics (n = 88)	Diabetics (n = 64)	p-value < 0.001
Mean Creatinine pre-PCI (mg/dL ± SD)	1.05 ± 0.23	1.07 ± 0.35	
Mean Creatinine post-PCI (mg/dL ± SD)	1.05 ± 0.25	1.12 ± 0.48	

## Discussion

The aim of our study was to investigate the incidence of CIN among the patients undergoing PCI at tertiary cardiac center of Nepal. In general, CIN following PCI is the third most common cause of AKI [4].

The true incidence of CIN is difficult to assess because of the differences in definition of contrast agent nephropathy across the various studies, the proportion of high-risk patients, the types of contrast media used, and the use of preventive measures. However, the scope of CIN is clearly large. The first reported incidence of CIN in Nepalese patients undergoing PCI by Sharma et al. was 8.18% which is slightly lower as compared to our study [5]. While our study was limited by the sample size, it was consistent with two other epidemiological studies including 1826 and 1196 patients undergoing coronary angiography, where incidence of CIN was 14.4% and 11.1%, respectively [6-7].

The other two larger studies, which included 7586 and 8628 patients undergoing PCI, used different and less sensitive diagnostic criteria than those more commonly used for CIN and reported incidences of CIN of 3.3% and 16.5%, respectively [2, 8]. Few other studies found that the female gender was an independent predictor of CIN development after PCI and a marker of increase one-year mortality after CIN in patients with no underlying chronic kidney disease [8-9]. However, we were not able to observe this observation in our study as our study populations were predominantly comprised of male subjects. In two of the previous studies, 0.3% to 4.0% of patients with CIN required short-term hemodialysis unlike our result [10-11].

None of our patients actually required hemodialysis after development of CIN. The main reason for this observation could be that none of our patients had end stage kidney disease and also the severity of CIN was mild. Rihal et al. observed that when renal function is mildly impaired, the risk of CIN in diabetics was 4.1%, about twice that in patients without diabetes [2]. Our study is also parallel to Rihal findings; incidence being 21.8% among diabetics and 6.8% among non-diabetics (p = 0.001). Our study is also supported by Lautin et al. where diabetes patients with baseline normal renal function had higher risk of developing CIN [12].

A retrospective analysis of data from the Mayo Clinic PCI Registry also revealed significantly greater short-term mortality with CIN. Of the 7586 patients undergoing PCI, 254 patients (3.3%) experienced CIN, and the in-hospital mortality rate for these patients was 22% compared with a rate of 1.4% for the patients who did not develop CIN (p < 0.0001) [13]. In the same study, it was also found that diabetics more commonly developed CIN. Unlike Mayo clinic registry, present study showed higher CIN without any mortality as compared to previous findings from Mayo clinic as well as from Victor's observation where 0.5% of patients died due to CIN [14]. Further larger study is recommended to determine the risk factors and outcome of CIN in Nepalese population.

Our study has few limitations including small sample size, single study center, and limited study period.

## Conclusions

CIN is a common complication following PCI especially in diabetics. Despite the use of iodinated material we had similar incidence of CIN comparing the incidence of CIN among various radiocontrast compounds used to visualize vessels. There was no mortality and most importantly, none of the patients required hemodialysis in our study when compared to few available studies. A key step to reduce CIN is to identify patients at risk of CIN.
